# Geographic diversity of human liver cancers mirrors global social inequalities

**DOI:** 10.3389/fonc.2025.1565692

**Published:** 2025-05-16

**Authors:** Luis Cano, Fabien Foucher, Orlando Musso

**Affiliations:** Institut National de la Santé et de la Recherche Médicale (INSERM), Institut National de Recherche pour l'Agriculture, l'Alimentation et l'environnement (INRAE), Univ Rennes, Nutrition Metabolisms and Cancer, Rennes, France

**Keywords:** liver fibrogenesis, tumor progression, phenotypic diversity, hepatitis, alcohol misuse, alcohol use disorder, alcohol-related liver disease, MAFLD

## Abstract

Liver cancers show high interindividual and intratumor heterogeneity. Among them, hepatocellular carcinoma (HCCs) represents approximately 90% of liver cancers, followed by intrahepatic cholangiocarcinoma (iCCA; ~10 to 15%), childhood hepatoblastoma, angiosarcoma and hemangioendothelioma (< 1%). More than 80% of HCCs arise in a backdrop of chronic inflammatory liver diseases of diverse etiologies. These underlying liver diseases are major determinants of geographic diversity of HCCs. Across the world, substantial differences in the prevalence of chronic viral hepatitides, alcohol misuse, Metabolic Disfunction-Associated Steatotic Liver Disease (MASLD) and exposure to toxic substances are frequently related to social and economic inequalities. Vulnerable populations are more frequently exposed to infections such as hepatitis B and C viruses that, combined with other risk factors, lead to both vertical and horizontal transmission and, in turn, impact on age and sex-related diversity. In this review, we describe the global landscape of risk factors leading to HCC: MASLD, chronic hepatitis B and C infections, alcohol misuse, exposure to other toxic substances and genetic predispositions. We describe their combined effects on the clinical and epidemiological features of HCCs around the globe. Clinical presentation, incidence and mortality rates of HCCs show therefore great geographic heterogeneity, which is also related to the inequalities in the gross domestic product *per capita*, the socio-demographic index, the access to health care resources and to the implementation of policies for surveillance and screening of patients at risk. Awareness of the biological and geopolitical sources of HCC diversity will hopefully lead to more efficient international cooperation in the prevention and early management of chronic liver diseases and HCC.

## Introduction

With approximately 800,000 deaths per year, liver cancer is the third deadliest cancer worldwide. According to the major cell type involved, liver cancers are classified as hepatocellular carcinoma (HCCs; ~90%), intrahepatic cholangiocarcinoma (iCCA, ~10 to 15%), childhood hepatoblastoma, angiosarcoma and hemangioendothelioma (< 1%). HCC occurs in over 80% of cases in the context of chronic fibroinflammatory liver diseases, and its heterogeneity complicates patient management (1). Despite advances in vaccination against hepatitis B virus and direct antivirals against hepatitis C virus, the incidence of HCC is increasing due to metabolic dysfunction-associated steatotic liver disease (MASLD), related to overweight, obesity, sedentary lifestyles and important changes in nutritional behaviors across the world (2, 3).

Therapeutic targeting of immune checkpoints has expanded treatment options for HCC; however, a response rate of 30% underscores the need to characterize tumor heterogeneity to better predict treatment response and target prescription (4). Surveillance for HCC emergence in patients with chronic liver diseases relies on biannual ultrasound or magnetic resonance imaging to detect small early-stage tumors that are candidates for potentially curative therapies (local ablation, resection or transplantation). However, high (up to 70%) 5-year recurrence rates after HCC resection or even higher after percutaneous ablation make liver transplantation the best possible treatment, with a recurrence rate of 10% (5). Approximately 80% of HCCs have metastases and/or local extension at diagnosis, limiting the access of patients to potentially curative therapies (4). Also, 30% to 40% of HCCs exhibiting clinical and molecular signs of good prognosis eventually recur within two years of resection (6, 7). HCC results from combined effects of comorbidities that lead to fibrogenesis, clinical signs of impaired liver function, and concomitantly, development of pre-cancerous and cancerous liver nodules (5). Some of the synergistically interacting comorbidities that contribute to HCC include social vulnerabilities and/or psychopathological conditions that result in alcohol and/or intravenous drug misuse. Also, as we will discuss in detail below, the epidemiology as viral hepatitides and MASLD is subject to significant geographic diversity that mirrors global social inequalities (8).

Therefore, the aim of this review is to raise awareness on the biological and socioeconomic sources of HCC diversity. This awareness will hopefully lead to more efficient international cooperation in the prevention and early management of chronic liver diseases and HCC.

To fulfill this aim, we first describe data sources and methods applied to construct this review, which are followed by a brief introductory overview on the epidemiology of liver cancer and on the pathophysiological features of HCC and iCCA. Then, we review the impact of socioeconomic diversity on liver cancer incidence and mortality and on the global landscape of risk factors. These concepts are further developed with an in-depth description of the clinical and epidemiological landscape of liver cancer in different world regions. Finally, we outline efforts across the world to improve surveillance of at-risk patients.

## Data sources and methods

Data presented in this article are mainly based on the GLOBOCAN database compiled by the Global Cancer Observatory (GCO), coordinated by the International Agency for Research on Cancer (IARC). When appropriate, data are completed and/or compared with those issued from the Global Burden of Disease (GBD) study from the Institute for Health Metrics and Evaluation. In addition, when trends require to delve deeper on specific epidemiological or pathophysiological points, we refer to independent studies published in peer-reviewed scientific literature indexed in PubMed. Given that HCCs represent 90% of liver cancers, publicly available international registries like GLOBOCAN and the GBD study lump together different histological types of primary liver cancers. Thus, except when indicated otherwise, data on epidemiological trends, incidence and mortality rates apply to primary liver cancers, without distinction.

The GLOBOCAN database and the GBD study rely on different estimates. While both provide valuable information, being aware of their methodological specificities may help data interpretation and avoid pitfalls such as erroneous conclusions generated by sampling biases and/or reporting from inhomogeneous sources across the world.

GLOBOCAN uses population-based cancer registries, collecting data from specific geographic areas and providing cancer incidence and mortality. In countries where registries are absent or insufficient, GLOBOCAN uses statistical modeling estimates. These are based on IARC collaborations with national cancer registries worldwide. For example, when national data are not available, sub-national data with coverage > 50% are used. To accomplish this goal, IARC launched the Global Initiative for Cancer Registry Development, creating regional hubs with dedicated staff for cancer registration, which include the Caribbean, Latin America, Northern Africa, Central and Western Asia, Pacific Island, South East and South-Eastern Asia and Sub-Saharan Africa (9, 10).

While GLOBOCAN is primarily focused on cancer, the GBD study covers a wide range of health risks and outcomes. GBD uses a variety of data sources, including verbal autopsy (VA) reports, hospital records and surveys; hence integrating heterogeneous data from multiple sources. Thus, statistical modeling and standardization in the GBD study are designed to minimize the effect of biases and uncertainty in low- and middle-income countries, where data availability is often limited. VA reports are a key tool in the GBD study. Since many countries lack reliable registration systems, causes of death are missing. Thus, VA is used to determine causes of death and cause-specific mortality fractions in populations without complete registries. They are obtained by trained interviewers using a standardized questionnaire to collect information about signs, symptoms and demographic features of a recently deceased individual. The *SmartVa* open source algorithm has been developed to assign causes of death to VAs by applying clinical diagnostic gold standards (11).

While the GBD’s VA-based estimations offer valuable insights, it is important to interpret them with an understanding of their limitations. One significant limitation of this method is that VA data are less consistent than pathology reports. Consequently, VA reports might inadvertently combine data from primary liver cancers with liver metastases originating from extrahepatic cancers. A second caveat is the documented failure to reproduce analyses from VA data, which likely stems from discrepancies in data pre-processing and parameter weighting. This contingency highlights the importance of publishing in GitHub the necessary code to replicate the work (12). In view of these limitations, we emphasize the relevant caveats when appropriate. In some cases, these limitations are due to incomplete registries and sampling biases, which can arise from disparities in the material and human resources allocated to public health authorities worldwide. By drawing attention to these issues, we hope to highlight regions and risk factors that particularly require international collaboration.

## Global overview on the epidemiology of liver cancer

As HCC is the most common form of liver cancer (13), both GLOBOCAN 2022 and the GDB study report data for primary liver cancers regardless of the histological type. Thus, unless specifically referring to data on one liver cancer type in particular, we report data on HCC and iCCA taken together.

In 2022, liver cancer was the sixth most common tumor and the third cause of cancer-associated death in the World, after lung and colorectal carcinomas, according to the International Agency for Research on Cancer (IARC) (8, 9, 14) with a global age-standardized incidence rate (ASIR) per 100,000 individuals of 12.7 in males; 4.8 in females and an age-standardized mortality rate (ASMR) per 100,000 individuals of 10.9 in males and 4.1 in females. Liver cancer remains the most common cause of cancer-related mortality in males in Mongolia; in many African countries, such as Egypt, Sudan, Mauritania, Senegal, Sierra Leone, Liberia, Ghana, Burkina Faso, Niger, Gabon, as well as in South-East Asian countries like Thailand, Laos, Cambodia, Vietnam and Papua New Guinea. Respectively, male/female ASIRs per 100,000 individuals are the highest in East Asia, in particular in Mongolia (22.4/7.2), in South-East Asia (21.2/6.8), followed by Australia-New Zealand (10.4/3.1), North America (10.0/3.6), Western Europe (8.5/2.8), Western Africa (9.7/5.7) and South America (5.4/3.5).

**Figure 1:**
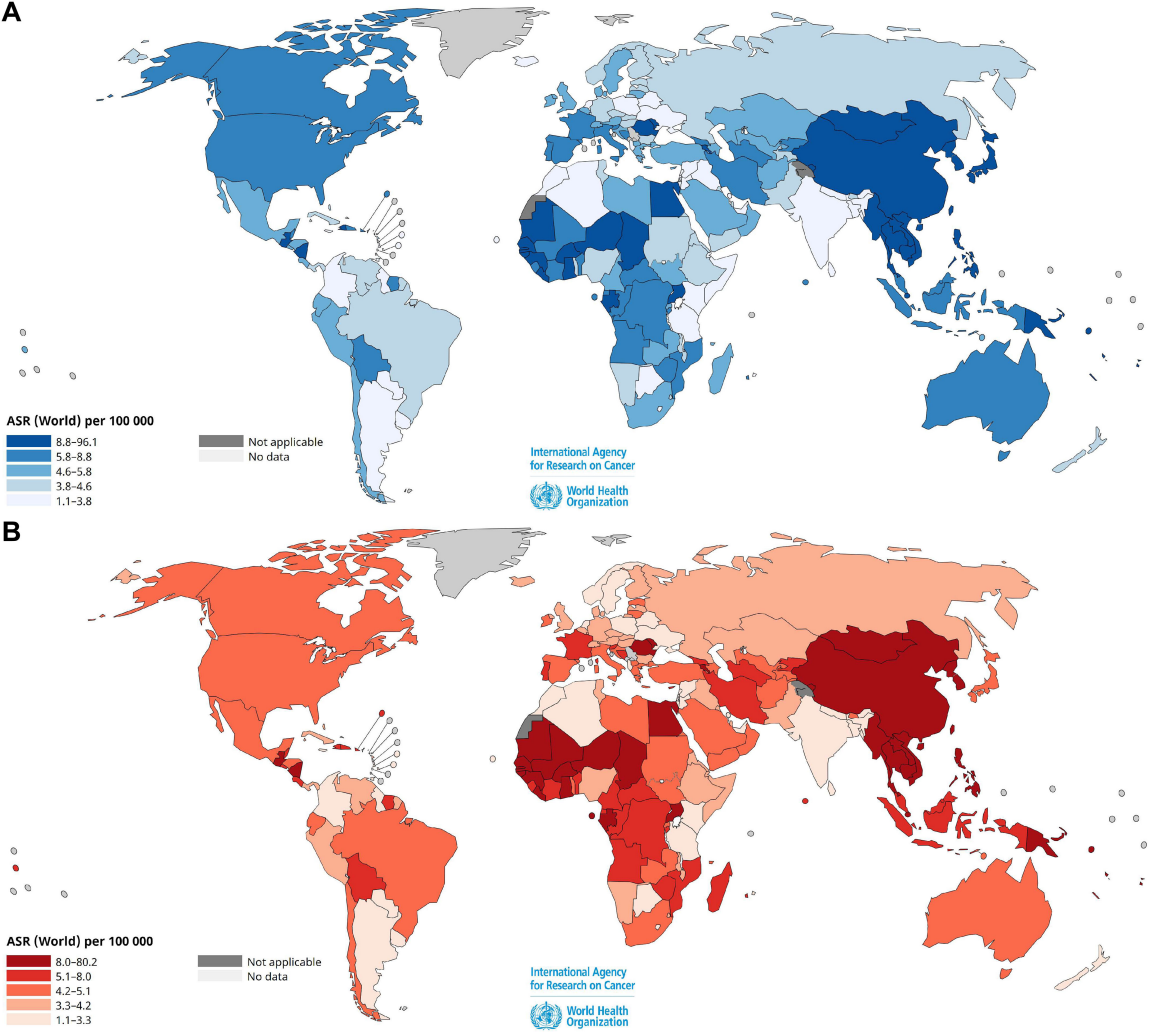
Estimated age-standardized incidence **(A)** and mortality **(B)** rates of liver and intrahepatic bile duct cancers worldwide per 10^5^ individuals, for both sexes in 2022. Data source: GLOBOCAN 2022, Cancer Today, International Agency for Research on Cancer, World Health Organization (https://gco.iarc.fr/en). Age-standardized rates (ASR) for 185 countries are shown in **Supplementary Table S1**.

**Figure 1** presents the estimated ASIRs and ASMRs of HCC and intrahepatic bile duct cancers worldwide per 100,000 individuals for men and women and **Supplementary Table S1** lists age-standardized rates for 185 countries, according to GLOBOCAN 2022. The Asia-Pacific region, Southeast and sub-Saharan Africa account for approximately 85% of the total liver cancer cases worldwide. China represents approximately 50% of all cases.

**Figure 2:**
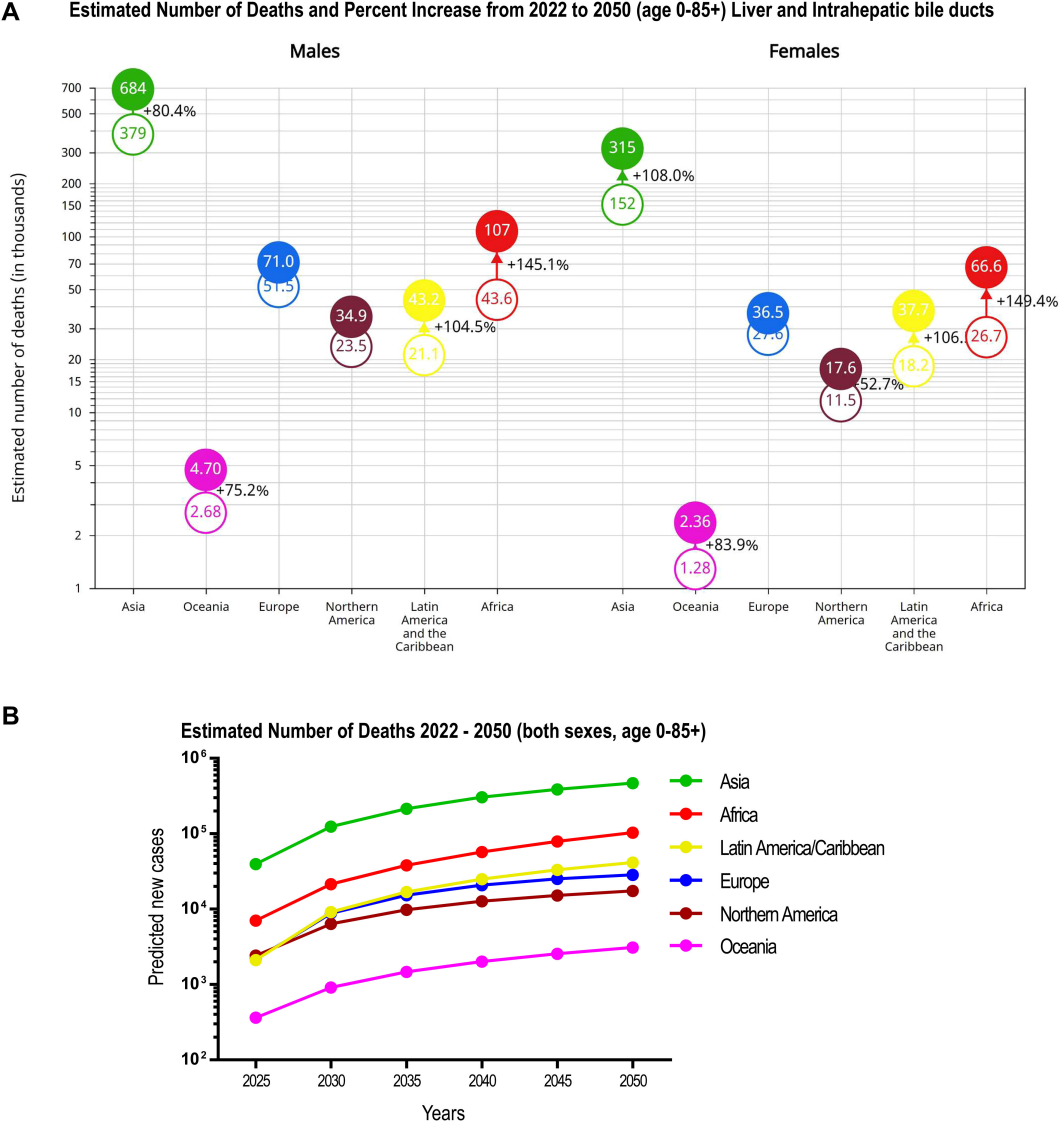
Estimated number of deaths and percent increase projection from 2022 to 2050 for liver and intrahepatic bile duct cancer. **(A)** Bubble chart showing estimated number of deaths and % increase in risk change. **(B)** Estimated timeline of new predicted cases of liver-cancer-related deaths (2025 through 2050) in five continents. Predicted numbers, risk and % change in five continents are shown in **Supplementary Table S2**. Data were obtained from the Global Cancer Observatory: Cancer Tomorrow, GLOBOCAN 2022, version 1.1 (August 02, 2024) (9). Data predict the future incidence and mortality from the current estimates in 2022 up until 2050, in 185 countries or territories grouped in five continents for both sexes. The key assumptions are that the national rates, as estimated in 2022, and the national population projections do not change throughout the 2022–2050 period. The expected number of new liver cancer-related deaths is calculated by multiplying age-specific mortality rates estimated for 2022 by the expected population for a given year extracted from the United Nations, World Population Prospects (2019 revision) (9).

According to the GBD study, liver cancer is responsible for approximately one million new cases worldwide, with 800,000 deaths (15). In view of this global trend throughout the last three decades, the World Health Organization (WHO) estimates that more than one million patients will die with a diagnosis of liver cancer in 2030 (16). In consistency with these data, **Figure 2A** shows the GLOBOCAN’s estimated number of deaths from 2022 to 2050 for HCC and intrahepatic bile duct cancer impacting both men and women. The highest increases are seen in Africa (+149% for women and +145% for men) and in Latin America and the Caribbean (+106% for women and + 104% for men). Notably, in Asia, a higher increase is predicted for women (+108%) than for men (+80.4%). A closer look at the estimated timeline of new predicted cases of liver cancer-related deaths from 2022 through 2050 in 185 countries from five continents (**Figure 2B**, **Supplementary Table S2**), according to GLOBOCAN 2022 (9), anticipates alarmingly steeper slopes of the curves after 2025, with the Latin America/Caribbean region approaching the trends of Asia and Africa. This epidemiologic modeling was done assuming that the national rates, as estimated in 2022, and the national population projections, do not change throughout the 2022–2050 period. Thus, the expected number of new liver cancer-related deaths was calculated by multiplying the estimated age-specific mortality rates in 2022 by the expected population for a given year extracted from the United Nations, World Population Prospects (2019 revision) (9). In consistency with these data, an independent analysis of the global burden of primary liver cancer in 2020 predicts a rise of >55% in 2040 (17).

## Pathophysiological features of hepatocellular carcinoma and intrahepatic cholangiocarcinoma

Although over the last years considerable progress has been made in the understanding of the molecular pathways driving liver carcinogenesis; early diagnosis, treatment, surveillance and prognosis of liver cancers remain major public health challenges worldwide. Notably, clinical and pathological features of liver cancers are subject to geographic variations in exposure to risk factors, at-risk patient surveillance, management and access to potentially curative treatments (8).

**Figure 3:**
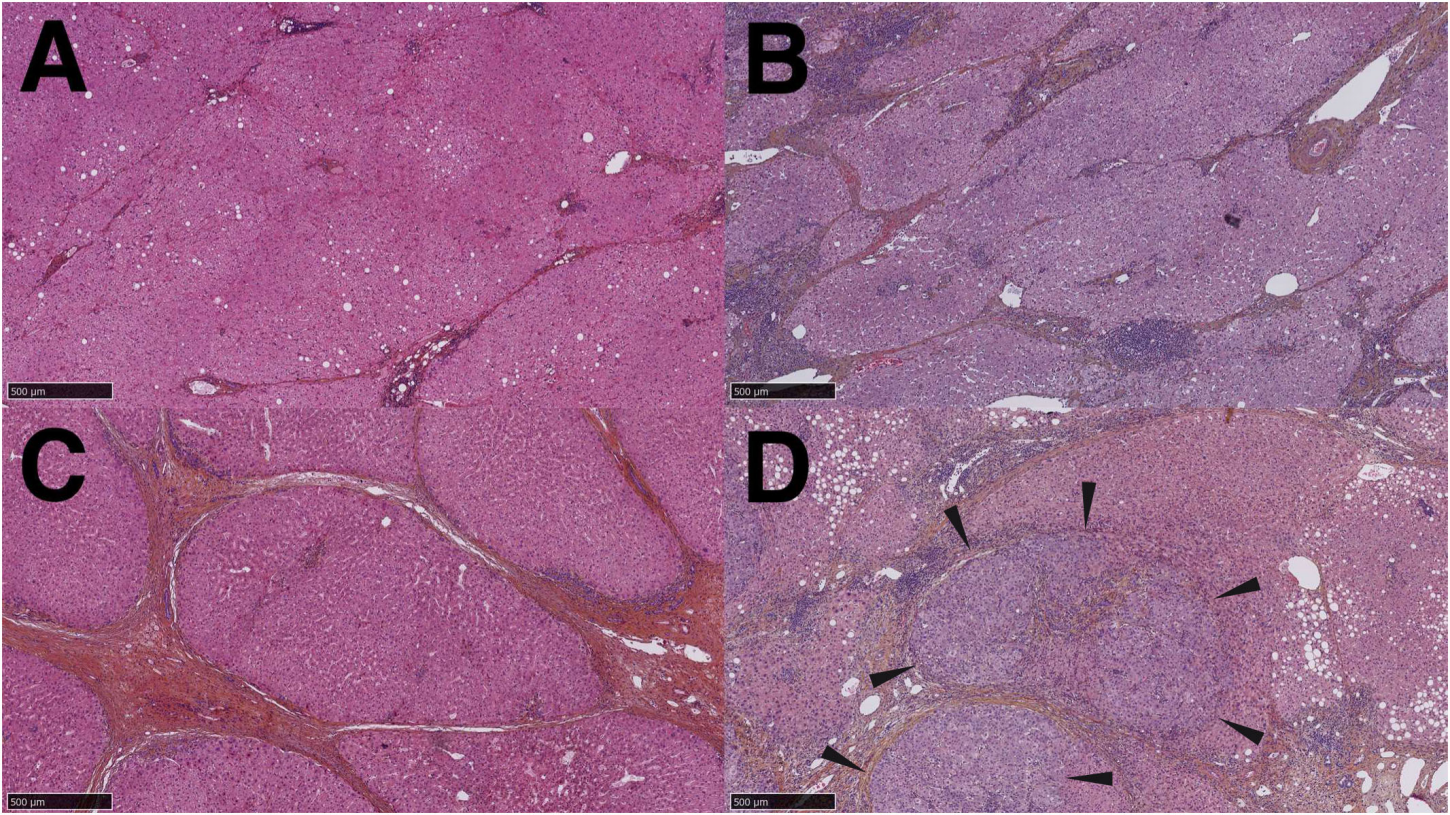
Stages of progression from chronic fibroinflammatory liver disease to hepatocellular carcinoma. **(A)**. Hepatic fibrosis stage 2, small and thin fibrous bands extend from the portal tracts. **(B)**. Stage 3 liver fibrosis, thick fibrous tracts partially outline regenerative nodules. **(C)**. Stage 4 liver fibrosis (cirrhosis), fibrous bands completely outline regenerative nodules. **(D)**. Hepatocellular carcinoma arising within a cirrhotic nodule: a tumor with architectural and cytological features of hepatocellular carcinoma is clearly distinguished from the surrounding parenchyma *(arrowheads)*.

HCC and intrahepatic cholangiocarcinoma (iCCA) have different pathogenesis; hence the modes of presentation, clinical, morphological and imaging features differ. A background of chronic fibroinflammatory liver diseases lead to repeated bouts of inflammation and hepatocyte death, followed by hepatocyte regeneration, fibrogenesis and ultimately to HCC (**Figure 3**). Over time, this process results in hepatocyte retro-differentiation and amplification of a contingent of bipotent epithelial liver progenitor cells, which promotes genomic instability and thus the emergence of preneoplastic and neoplastic foci. The lobular liver tissue architecture progressively changes into regenerative hepatocyte nodules outlined by fibroinflammatory septa, wherein biliary metaplasia of hepatocytes and ductular reaction are commonly observed. These architectural changes, together with sinusoidal capillarization, impair vascular flow and hepatocyte metabolic functions. Chronic inflammatory liver disease has been compared to a minefield; hence the term “field effect” to embody the risk of emergence of HCC in a backdrop of severe, life-threatening liver disease (18, 19).

Intrahepatic cholangiocarcinoma (iCCA) is a primary liver malignancy with increasing global incidence, particularly in Western countries. Major risk factors include chronic liver diseases (HBV, HCV, MASLD/MASH), primary sclerosing cholangitis, parasitic infections affecting the liver, and exposure to carcinogens. It involves key genetic alterations such as *FGFR2* fusions, *IDH1/2* mutations, *KRAS, TP53*, and *BAP1* mutations, although it shares with HCC inflammatory and fibrotic pathways promoting tumor progression. Like HCC, the diagnosis of iCCA relies on MRI and CT scans with contrast enhancement patterns; however, histopathology remains essential; in particular in cases where the differential diagnosis between HCC and iCCA is difficult on the basis of imaging features. Surgical resection is the only curative option, but it is feasible in less than 30% of cases. Like HCC, the prognosis of iCCA remains poor, with 5-year survival under 20%. Thus, early detection and molecular profiling are critical for improving outcomes (20, 21).

## Geographic and socioeconomic diversity versus ethnic origin in the appraisal of liver cancer heterogeneity

Applied to Humans, the concepts of “race” and “ethnic origin” are not substantiated by scientific data and they infringe legislation in some European countries. Definition of these populational categories on the basis of skin color and/or facies poses both an ethical and a scientific problem (22). Collection of ethnic data and definition of ethnicity by a third party raises fundamental human rights issues, such as the non-respect of the right of individual self-identification (23). Therefore, we focused this review as much as possible on the reported locations of patient care, that in most cases correspond to the nearest referral hospital to the patient’s dwelling-place. This approach has the advantage of being related to the patient’s exposure to xenobiotics, infectious agents, lifestyle and access to healthcare systems.

Self-reported ancestry respects the fundamental right of self-identification (23) and has a measurable impact on liver cancer epidemiological data. For example, Latin America has a mixed population from different ancestries based on waves of European colonization and African forced emigration through transatlantic slave trade during the 18^th^ century. Later on, in the 19^th^ and 20^th^ centuries, voluntary migration from Europe, Asia and the Middle East, led to high cultural and genetic diversity (24, 25). Thus, data from six countries in the ESCALON European-Latin American network prospectively following 429 hepatobiliary cancer patients (26, 27) showed important differences in liver cancer risk factors in self-reported European and non-European patients. These data will be discussed in the section addressing risk factors. Similarly, in North America, particularly in the USA, immigrants from Eastern, Southern, and Central Europe during the late 19^th^ to early 21^st^ centuries, and more recently from Latin America, Asia, and the Caribbean, have significantly shaped American society (28). While not legally classified as immigration, the transatlantic slave trade, which began in the early 17^th^ century and continued until 1865, played a crucial role in forming a diverse population with varied ancestries (29). Therefore, as discussed below, integrating analyses from cancer and risk factor registries with self-reported ancestry data offers valuable insights into how risk factors differently impact various communities in the USA (30, 31).

Analyzing the impact of population groups, ancestry, and geographic origin of immigrants on the incidence of etiology-specific HCC reveals two key insights: First, immigrants bring with them not only their genetic background but also risk factors associated with their original country or region. While the influence of these risk factors may diminish over time, interactions between past and current environmental exposures can result in unique combinations of risk factors. Second, categorizing patients based on skin color, language, or continent and country of origin highlights the complexity of defining population classes, particularly in the Americas. This complexity underscores the need for a more nuanced understanding of the interplay between genetic, environmental, and social factors in health outcomes (32).

## Socioeconomic diversity greatly impacts liver cancer incidence and mortality

Globally, in high/very high human development index (HDI) countries, liver cancer ASIR/ASMR per 100,000 individuals are 15.3/12.9 in males and 5.3/4.5 in females; whereas in low/medium HDI countries, liver cancer ASIR/ASMR are 6.2/5.9 in males and 3.1/3.0 in females (9). These data call for cautious interpretation, as ASIR/ASMR in low HDI regions are based on estimations from fragmented data, as pointed out above, and cannot be validated with the same robustness as data in high HDI regions. Rather, the take-home message here is that socioeconomic inequalities also impact the resources allocated to data collection leading to considerable heterogeneity (33).

In an effort to quantify the impact of social inequities, a socio-demographic index (SDI) has been used as a composite indicator of development status, which is strongly correlated with health outcomes. It is calculated as a geometric means of 0–1 indices of income *per capita*, average years of schooling over the age of 15 and total fertility under the age of 25. Thus, an SDI=0 indicates minimum and SDI=1 indicates maximum levels. SDI is often completed with the universal health coverage index (UHCI), which is reported on a 0–100 scale. It is based on indicators such as direct measures of therapeutic coverage, outcomes, such as mortality-to-incidence ratios and access to quality care (34).

Using the above indicators, a study based on the Global Burden of Liver Cancer (35) revealed an increasing trend in ASIR of primary liver cancer between 1990 and 2019 in nearly half (91/204) of the countries and territories included in the Global Burden of Disease (GBD) study. Surprisingly, using Pearson correlation analysis, the authors detected a low but significant positive correlation of SDI with an increase in ASIR (ρ=0.31) and in ASMR (ρ=0.26) of primary liver cancer among countries or territories with an SDI ≥ 0.7. No correlation was found in regions with SDI < 0.7. Similarly, the authors revealed a significant positive correlation of an increase in ASIR (ρ=0.49) and in ASMR (ρ=0.47) of primary liver cancer among countries or territories with an UHCI ≥ 70. No correlation was identified with UHCI <70. The authors explain this trend by the contribution of immigrant minorities in high SDI Western countries, as seen in the USA, Australia and Canada, where the highest incidence of liver cancer was identified among immigrants from high-risk countries (35). However, and as pointed out above, data issued from high *versus* low socioeconomic development regions of the world need cautious interpretation, as they are biased by the heterogeneity of the sources feeding estimation models (33). Conversely, registry-based studies in the USA showed that Latin American foreign-born patients with HCC (36) or liver cancer (37) had better survival than US-born patients, and that increasing generational status of Mexican Americans in the USA was associated with a higher risk of HCC (38), suggesting that USA birthplace is a risk factor for liver cancer death in Mexican Americans.

A meta-analysis of 20 studies between 1994 and 2024 including cross-sectional, cohort and case-control studies showed that patients in disadvantaged areas faced delayed treatments and worse outcomes (39). Similarly, a study involving 1,485 patients in China showed that low household income was independently associated with advanced disease and poor outcome in patients with HCC (40).

**Figure 4:**
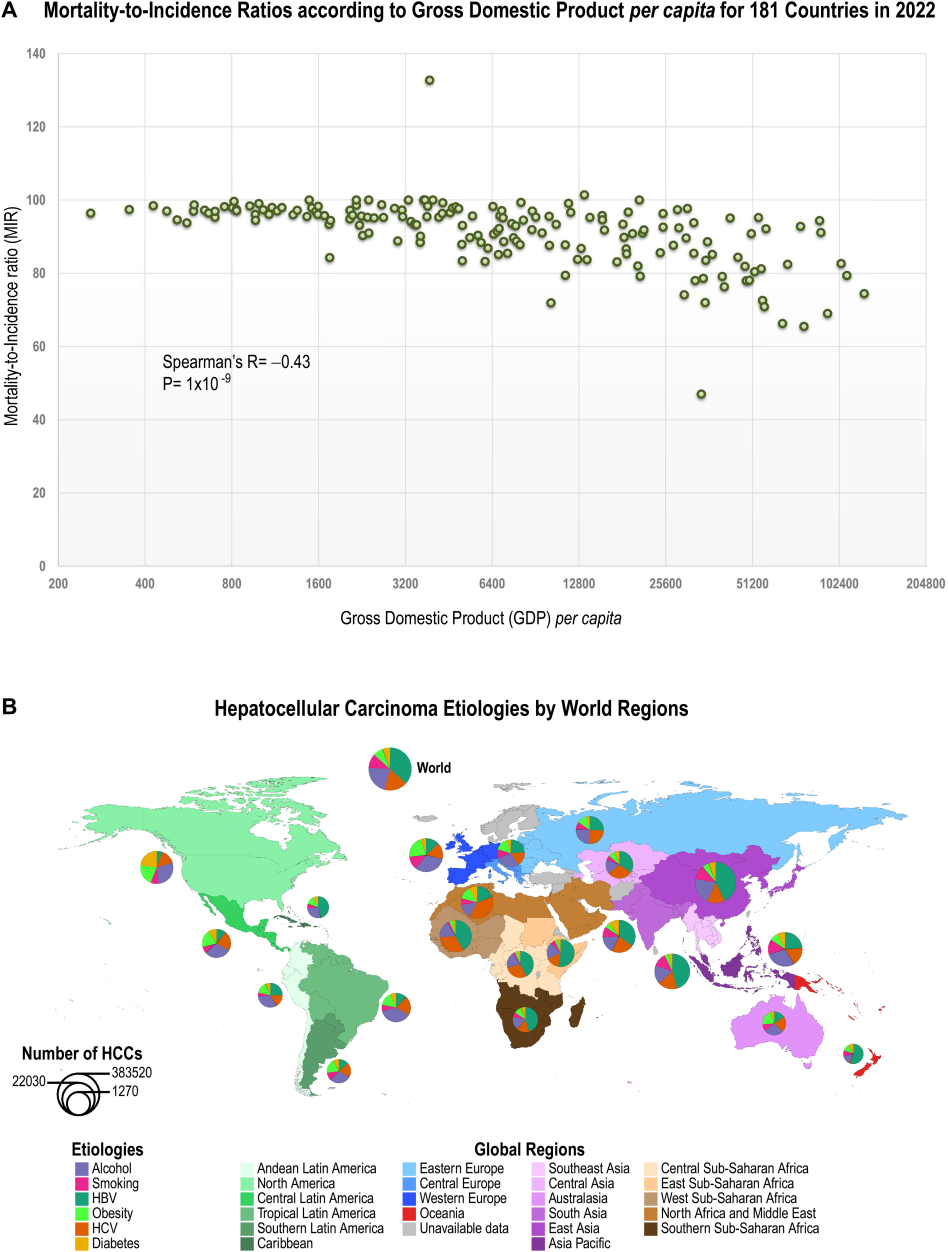
Socioeconomic diversity greatly impacts liver cancer incidence and mortality. **(A)** Relationship between Mortality-to-Incidence ratios (MIRs) and Gross Domestic Product (GDP) *per capita* for 181 countries in 2022. MIRs for each country= (ASR mortality per 10^5^ individuals/ASR incidence per 10^5^ individuals) x 100. GDPs and MIRs for each country, obtained from GLOBOCAN 2022, are shown in **Supplementary Table S3**. GDP is the sum of gross value added by all resident producers in the economy plus any product taxes and minus any subsidies not included in the value of the products. GDPs *per capita* for the year 2022 in current US$ is the GDP divided by midyear population. Publicly available GDPs *per capita* were obtained from the World Bank Group under the Creative Commons Attribution 4.0 International license (CC-BY 4.0). The source of the raw data are World Bank national accounts and OECD National Account data files. **(B)** Fractions of hepatocellular carcinoma etiologies by World regions (41). Regions, continents and etiologies are color-coded. *“Number of HCCs”* indicates the approximate number of HCC cases studied, which is proportional to circle radius. Circle radius, r= ln (HCC number) x 0.7; *i.e.*, natural logarithm of HCC number x 0.7. Quantitative data for the 21 World regions are shown in **Supplementary Table S4**.

We thus studied the relationship between Gross Domestic Products (GDPs) *per capita* and the Mortality-to-Incidence Ratios (MIRs) for liver cancer in 181 countries in 2022. We found that the MIRs for liver cancer were inversely proportional to the GDPs *per capita.* That is, the higher the GDP, the lower the MIR (**Figure 4A**). MIRs and GDPs in current US$ for 181 countries in 2022 are listed in **Supplementary Table S3**. GDPs were obtained from the World Bank Group.

The impact of poverty is not exclusive to low-income countries. In France, higher HCC incidence is related to an unfavorable socioeconomic environment (42). This evidence highlights the interactions between social inequities and the aforementioned pathological background of comorbidities leading to HCC: alcohol and intravenous drug addictions, unchecked viral infections, as well as imbalanced nutritional and physical activity lifestyles. These risk factors are reviewed in detail in the next section.

## Global landscape of risk factors associated with liver cancer

The Population Attributable Fraction (PAF) is the proportion of a disease in a population that can be attributed to a specific risk factor. It estimates the potential reduction in the outcome if the risk factor were eliminated, assuming a causal relationship. PAF is calculated using the prevalence of the risk factor and its relative risk. It helps prioritize public health interventions by identifying the impact of reducing exposure to modifiable risks on overall population health.

**Figure 5:**
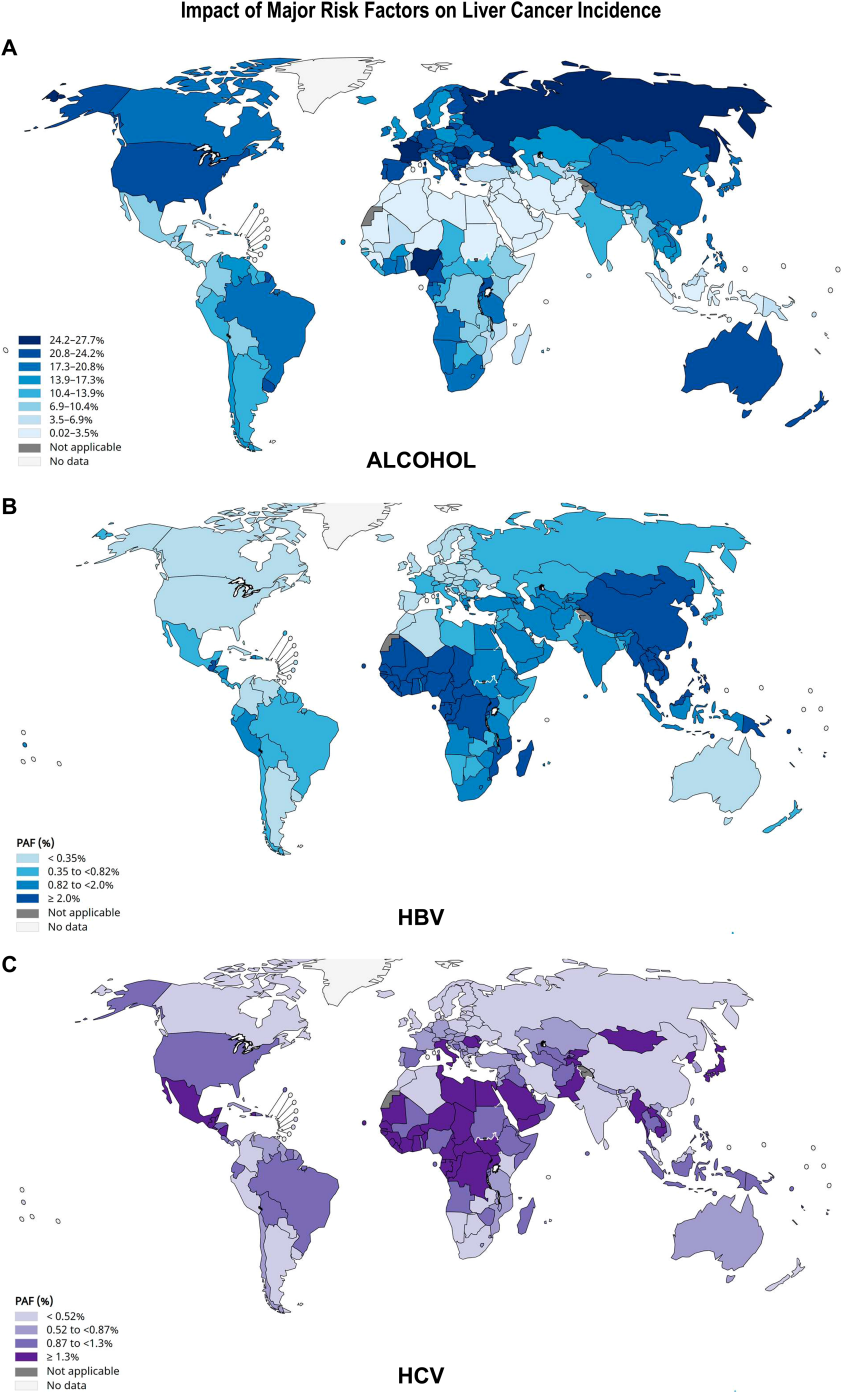
Impact of alcohol, HBV and HCV infections on liver cancer incidence. The population attributable fraction (PAF) is the proportion of cases of liver cancer that could theoretically be avoided if exposure to **(A)** alcohol; **(B)** HBV or **(C)** HCV was removed. Alcohol-related risk was modelled with an upper integration limit of 150 g per day. The authors also estimated the contribution of different levels of alcohol drinking by categorizing consumption into three levels: moderate (0.1–20 g/day; risky, 20–60; heavy, >60 g/day) (43). For HBV and HCV, the number of new cancer cases attributable to each infection was calculated by multiplying incidence estimates by the PAF, according to (44). The scales of color shades on the left are based on an absolute method, each group has the same number of observations. Source: GLOBOCAN 2022.

**Figure 4B** presents an overview of HCC etiologies by World regions and quantitative data for 21 World regions are shown in **Supplementary Table S4** (41). **Figures 5A-C** shows World maps with the population attributable fractions of liver cancer by country related to alcohol, HBV and HCV (43, 44).The study of the fraction of liver cancer cases attributable to five major risk factors by geographic region and gender showed that 44% of the World total liver cancer cases were attributable to HBV, 21% to HCV; 26% to alcohol, 13% to tobacco smoking, 9% to obesity and 7% to diabetes (**Supplementary Table S4**) (41). By contrast, subnational studies in the USA, covering either Florida or California, and based on actual cancer and hospital discharge registries, as well as serologically proven HCV infections, report that over 40% of all HCC cases are attributed to HCV (30, 31). As shown in **Figure 5**, PAFs are subject to important geographical variations. Below, we present a detailed overview of the global landscape of the major risk factors leading to liver cancer.

### Alcohol

Chronic alcohol consumption can be categorized into three levels: moderate (0.1–20 g/day; risky, 20–60; heavy, >60 g/day) (43). Numerous studies indicate that, in different concentrations, alcohol has harmful effects on health. A meta-analysis published by Corrao et al. (45) showed that with daily alcohol intake > 25 g/day, there is a relative increase in the development of chronic liver disease. IARC also indicates that higher amounts have direct hepatocarcinogenic effects (46).

Global data on alcohol misuse vary according to the definition of geographic locations and the amounts of alcohol intake considered: 6% in the Middle East; up to 14% in North Africa, between 50% and 60% in Eastern Europe, whereas in Southern Europe (Spain and Italy), the prevalence is about 20% (47). According to the situational report on alcohol consumption published by the WHO in 2016, the highest rate of active drinkers was found in Europe, with 60% of the total population > 15 years old; followed by the Americas with 54.1%, the Western Pacific area with 53.8%, South East-Asia with 33.1% and finally Africa with 32.2% (48).

Alcoholic liver disease (ALD) comprises a spectrum of conditions from reversible fatty liver to acute alcoholic hepatitis, chronic inflammation, fibrosis and cirrhosis, a late stage of disease during which HCC often develops. ALD is a major cause of HCC worldwide (47). In ALD patients, the diagnosis of HCC is often delayed with symptomatic cases at presentation. Patients present with a poorer general condition, more severely impaired liver function and higher prevalence of comorbidities. However, when HCC is diagnosed during surveillance in ALD patients, the rate of allocation to first-line curative treatments is high; although it has to be considered that in these patients there are higher surveillance failure rates; in part related to decreased sensitivity of ultrasound screening. Notably, patients with moderate alcohol consumption (≤ 60 g/day) associated with one or more metabolic risk factors exhibit an aggravated HCC risk profile due to the synergistic effect of MASLD and alcohol (49). Similar results were reported by a worldwide meta-analysis including 86,345 patients, showing that 30.4% of HCCs were ALD-associated, with the highest proportion in Europe and the lowest in the Americas. ALD-related HCCs had a more advanced BCLC (Barcelona Clinic Liver Cancer) stage and higher mortality rates when compared with other causes (50).

The importance of ALD-related HCC led to the development of statistical methods to compare the impact of worldwide public health policies on ALD prevention. Thus, an alcohol preparedness index (API) was recently proposed in a multi-national study including 169 countries between 2010–2019; analyzing data from the Global Burden of Disease database. High APIs were inversely correlated with alcohol use disorder, alcohol-related liver disease mortality and alcohol-attributable HCC. The highest associations were found in the Americas, Africa and Europe. Importantly, alcohol-attributable HCC incidence decreased after 8 years from baseline assessment. Thus, liver cancer-related mortality in regions with API ≤ 10 was about 15 cases per 100,000; whereas in regions with API=100, it was about 3 cases per 100,000 (51).

ALD prevention also involves alcohol rehabilitation to reduce ALD-related HCC risk. The effect of alcohol rehabilitation and abstinence on cancer incidence was assessed in people with alcohol dependence in a nationwide retrospective cohort study. Among 24 million patients discharged from French hospitals between 2018–2021, alcohol dependence was identified in 6.3% men and 1.6% women and was strongly associated with liver, oral, pharyngeal, laryngeal, esophageal and colorectal cancers. As expected, rehabilitation treatment or abstinence was associated with lower cancer risk (52).

Although most Western European countries benefit from the highest worldwide APIs, there is room for improvement, at least in France. In 2022, an average of 165 g of pure alcohol per week per individual over 15 years of age was sold in France. This is 1.65-fold higher than the upper limit of “reasonable” alcohol consumption recommended by health authorities. The documented impact of alcohol rehabilitation on cancer risk reduction suggests that most individuals, although aware of the health risks related with alcohol misuse, are not aware of their own risk. The reason for this cognitive bias could an underestimation of the amount and frequency of alcohol intake. In France, despite a high API, the information on ALD prevention that the media deliver in compliance with Public Health regulations is stigmatizing (53) and lacks precision: *“alcohol abuse is dangerous for your health”* and *“drink with moderation”.* These slogans lead the population to a complacent self-definition of “moderation” and “frequency” of alcohol consumption and, ultimately, to a lack of self-awareness of alcohol misuse and alcohol addiction (54).

The above evidence leads to the core problem of ALD, which is alcohol use disorder (AUD), as defined by the *Diagnostic and Statistical Manual of Mental Disorders, 5^th^ Edition (DSM-5)*, that lists 11 behavioral criteria for diagnosis and assessment of the severity of AUD. Still, AUD can be difficult to diagnose because many patients do not disclose alcohol use, frequently through lack of self-awareness or denial, and remain oligosymptomatic with subclinical but progressive disease (55).

Taken together, alcohol misuse is a cofactor in the development of chronic liver disease, fibrogenesis and liver cancer. ALD increases the harmful effects of other comorbidities, such as viral hepatitis and/or MASLD. In particular, the growing incidence of MASLD and combined ALD-MASLD worldwide call for public health policies focusing on the social determinants of alcohol dependence and nutritional behavior disorders.

### Other toxic substances

Other risk factors not necessarily related to a chronic inflammatory background and fibrogenesis are associated with HCC development, such as tobacco smoking, exposure to genotoxins like aflatoxins and aristolochic acid through the consumption of agricultural products (56–58). Exposure of patients to these chemical compounds can be suspected by the detection of typical mutational signatures in HCCs (59).

The association of tobacco smoking ranges from 4% to 14%, with the highest values in Southeast Asia (**Supplementary Table S4**) (41). Cigarette smoking is associated with a 70% increased risk of liver cancer (60)**;** the risk progressively decreasing and effectively disappearing after 30 years of tobacco arrest (61). Several studies have identified tobacco smoking as an independent risk factor for liver fibrosis, thereby contributing to liver carcinogenesis. In the liver, smoking leads to inflammation, insulin resistance and tissue hypoxia. Smoking is also seen as an aggravating cofactor in patients with chronic hepatitis, obesity and/or alcohol misuse (62).

Aflatoxins are mycotoxins that show strong hepatocarcinogenic effects. They are produced by *Aspergillus parasiticus* and *Aspergillus flavus* fungi that grow in staple cereals and oilseeds stored under favorable moisture and temperature conditions. Aflatoxin contamination is widespread in tropical and subtropical areas around the World, such as Southeast Asia and Sub-Saharan Africa (63, 64).

Four major types of aflatoxins are known: B1, B2, G1 and G2. The B1 aflatoxin (AFB1) is the most potent liver carcinogen (65). The mechanism associated with carcinogenic effects of AFB1 is DNA adducts formation, which cause a mutation (AGG to AGT) in codon 249 in the tumor suppressor gene TP53, resulting in substitution of arginine for serine (R249S) (66).

In Europe, the European Prospective Investigation into Cancer and Nutrition study cohort consisted of 521,323 adults throughout 23 centers in 10 European countries. This study found an association between deoxynivalenol (a mycotoxin produced by Fusarium species of fungi) and HCC risk. Deoxynivalenol can be found in cereal grains and products and derived thereof, such as wheat, barley, maize, oats and rye. However, this study did not statistical associations of HCC risk with the other mycotoxins in Europe (67).

Aristolochic acid (AA) is an abundant compound found in plants of the genders *Aristolichia, Bragantia* and *Asarum*, which are commonly used in traditional Chinese medicine (68). Several studies showed a relationship between AA and the development of urothelial and renal carcinomas (69, 70). Furthermore, AA is responsible for liver cancer development as demonstrated in Taiwan and in Asia (71). A possible mechanism explaining this association is that metabolites of AA could bind purines, thus contributing to the formation of adducts that result in transversion of adenine-to-thymine mutations (72, 73). However, other chemical compounds have similar mechanisms of carcinogenesis, such as vinyl chloride, 4-aminobiphenyl 1,3-butadiene, ethylene oxide (74, 75).

### Hepatitis B virus infection

HBV is a virus that contains a partial double strand of DNA belonging to the family of *Hepadnaviridae* (76). It was estimated that 1 in 3 people in the World had been infected with HBV (77), of which only 5% would develop chronic carrier status (78). HBV was responsible for at least 50~80% cases of HCC worldwide (79), mainly because chronic HBV infection is endemic in developing countries owing to suboptimal resources to implement prevention policies (80). HBV ranges from 6% to 14% in North and South America, with 24% in Andean Latin America; 12% in Western Europe; 39% in Eastern Europe to 50–80% in Eastern Asia, Sub-Saharan Africa and Oceania (**Figures 4B**; **5B**; **Supplementary Table S4**). Custer et al. (81) classified countries according to the prevalence rates of HBV infection:

- Low, <2% positivity for surface antigen B (HBsAg) in countries such as North America, Northern Europe, Australia and New Zealand.
- Intermediate, 2-7% of HBsAg positivity in Japan, the Middle East, Eastern Europe, Southern Europe and some areas in South America.
- High, > 8% HBsAg positivity in sub-Saharan Africa, the Amazon Basin, China, Korea, and Taiwan.

There are also important differences regarding the presentation of HBV infection and the geographic location. For example, in Africa, higher prevalence rates are reported in rural compared to urban areas (82). This diversity also extends among different African countries; for example in Burkina Faso, the estimated prevalence is 17.3% (83), compared to Cameroon, where the prevalence of HBV is 10.1% (84). Altogether, over the last 30 years, a high risk of contracting HBV by children has been described in Africa, in particular in rural areas with different socioeconomic conditions (85, 86).

At the molecular level, there are 5 viral genotypes (A, B, C, D, and E) (82, 87) most frequently described. A geographic distribution associated with genotypes also emerges, highlighting genotype A in the South East of Africa and in some regions of Northern Europe. Genotype D shows a predilection for Northern Africa, as well as southern Europe, including countries such as Italy, Greece, Serbia and Montenegro. Thus, high levels of incidence and prevalence were found in Syria, Iran, Turkey, Iraq, Mongolia, Kazakhstan, Russia and India (88). Genotypes B and C were the most frequent in China and also the Asia pacific region (89), whereas genotype E was prevalent in Africa, from Senegal to Namibia (87, 90, 91).

Despite geographic heterogeneity, global HBV immunization programs show favorable results, which gives a more encouraging outlook on the foreseeable future. In the 21^st^ century, China has seen a decrease in HBsAg carriers from 9.8% to 7.18% (92–94). However, there was still a broad breach to cover since the program had only protected 20% of rural areas. In South Korea, seroprevalence was 4%, with a reduction of chronic infection rates from 2.2 to 0.12% (95, 96). India showed seroprevalence rates of 3.1% in non-tribal populations and 11.85% in tribal populations (97, 98). Likewise, in Southwestern Asia, mainly in the territories of the Arabian Peninsula, rates ranged from 1.5% to 8% (99, 100).

An even more florid reality is found in Latin America, where it has been estimated that between 7–12 million people were chronic HBV carriers (101, 102). HBV infection rates are particularly alarming in the Amazon basin. This region extends over 8 countries: Perú, Brazil, Colombia, Bolivia, Ecuador, Venezuela, Guyana and Surinam (103), where it has been estimated that the HBsAg infection rate was greater than 8%. A study on 37 Peruvian aboriginal communities distributed in 12 areas reported infection rates of 59.7% in patients whose mean age was 22.7 years (102). Notably, combined vertical and horizontal transmission result in high rates of HBV infection in children under 10 years of age, reaching 82.5% positivity in patients over 45 years (104, 105).

The WHO’s global hepatitis strategy aims to reduce hepatitis seroprevalence by 90% and related deaths by 65% by 2030. By the end of 2022, HBV vaccine had been applied in 190 states with a global coverage of 84% for three doses. With the decline in the seroprevalence of HBV and HCV infections, as well as aflatoxin exposure, liver cancer rates have been declining in high-risk countries within the last 50 years. By contrast, incidence rates in formerly low-risk countries have increased in recent years because of the increasing prevalence of MASLD and ALD (9).

### Hepatitis C virus infection

**Figure 6:**
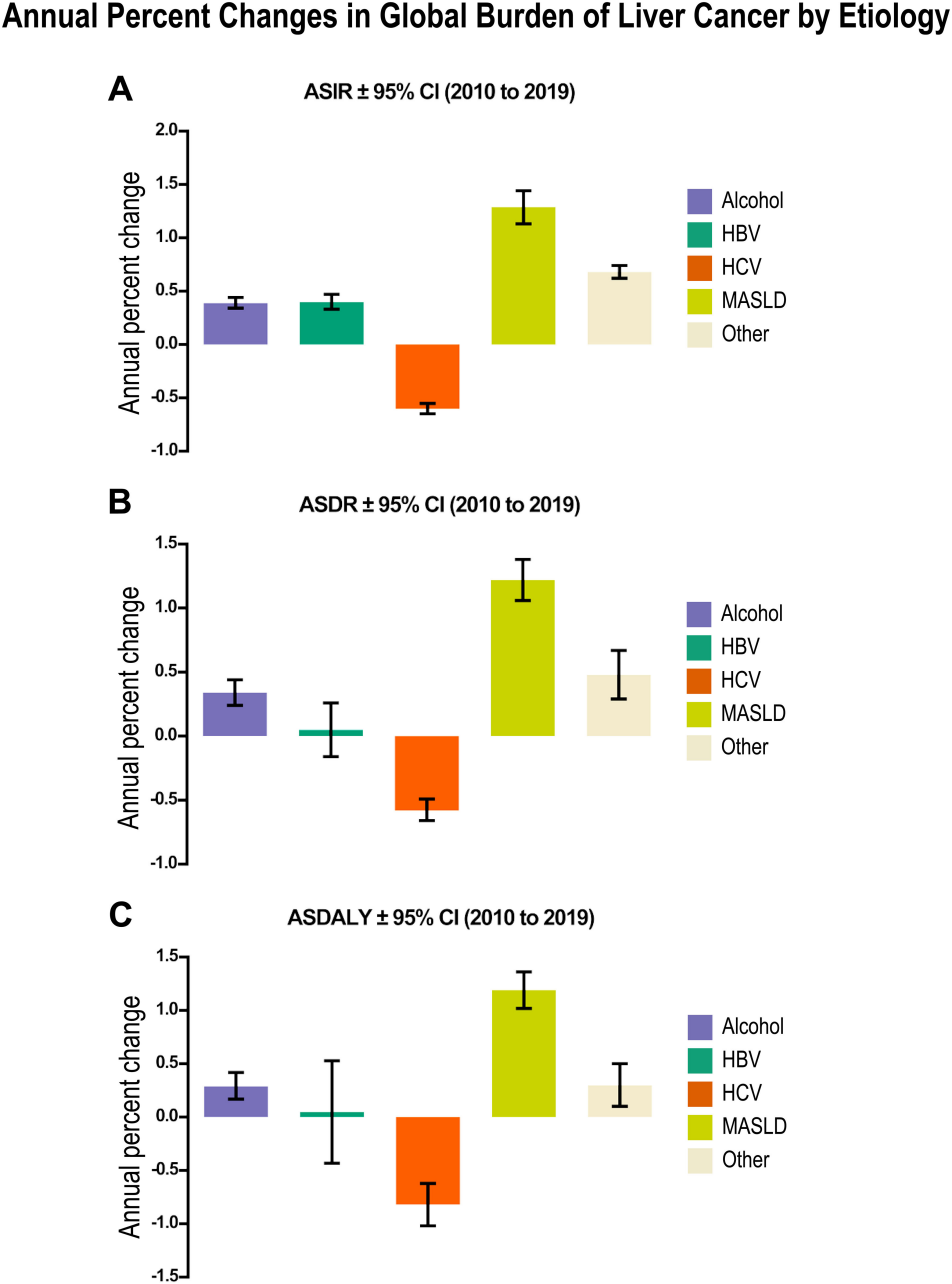
Annual percent changes in global burden of liver cancer by etiology from 2010 to 2019 in men reveals a major impact of MASLD in liver cancer incidence, death and patient disability. **(A)** Age-standardized incidence rates (ASIR) per 1x10^5^ ± 95% CI of liver cancer. **(B)** Age-standardized death rates (ASDR) per 1x10^5^ ± 95% CI of liver cancer. **(C)** Age-standardized disability-adjusted life-years (ASDALY) per 1x10^5^ ± 95 CI related to each etiological factor. Relevant tabular data extracted from (111) are presented here in chart form.

Hepatitis C virus is a single stranded RNA virus, which was identified in 1989 (106). As shown in **Figure 5**, despite the advent of direct antiviral agents (DAA), HCV is still considered a public health problem worldwide and a major cause of chronic liver disease and HCC in most developing countries and most regions of USA (107, 108). Thanks to the use DAAs, the global estimate of the prevalence of HCV decreased from over 100 million in 2015 to 58 million infected adults in 2019 (108–110). This evolution explains in part the decrease in the global HCV-related liver cancer burden (**Figure 6**). HCV PAFs range from 50–56% in Central Asia, West Sub-Saharan Africa, North Africa and Middle East, through 39% in Eastern Europe, to 15–20% in Western Europe, North and South America (**Figures 4B**; **5C**; **Supplementary Table S4**). However, the estimated 15-20% PAFs for North America were recently challenged by data from actual cancer registries. These registries, linked to hospital discharge agencies and viral hepatitis departments, indicate that over 40% of all HCC cases are attributed to HCV, according to recent studies in Florida and California (30, 31).

In the pre-DAA era, disparities in the prevalence of HCV infection were however revealed when comparing high-income countries (HIC), where the prevalence was generally below 2% (112), with low-middle income countries (LMIC), where the prevalence was higher than 5% like, for example, in Egypt, 4.4-15% (113), Cameroon, 4.9-13.8% (114), and Mongolia, 9.6-10.8% (115).

Blood transfusions played a significant role in the spread of HCV infections, particularly when blood screening practices were not yet optimal. For example, the risk of being infected with HCV in a blood transfusion was 2.5% per 1000 units in sub-Saharan Africa (116). Also, high-risk behaviors such as sharing syringes and needles by intravenous drug users were responsible for high transmission rates, with 1.8 to 46.7 cases of HCV infection/100 people/year, mainly in people inside prisons or detention centers. Finally, other potential forms of transmission could be: intranasal use of cocaine, tattooing, body piercings, sexual intercourse with blood exchange and cupping (56).

HCV does not integrate into the host genome (117) and constantly replicates during chronic infection. Genetic studies have shown that polymorphisms or mutations in the genes LEPR, MICA/HCP5 and IFNL3 loci (118–122) are more likely to develop HCC. In particular, mutations in the IFNL3 gene present a particular geographic distribution pattern, with lower frequencies in African and higher in Asian populations (123, 124).

Another factor is the genetic makeup of HCV itself. Certain HCV genotypes have shown a strong association with the development of HCC: the genotype 3a is mostly associated with HCC in a MASH context (125), whereas the genotype 1b is associated almost exclusively with a more frequent progression towards HCC (126). Geographically, the different genotypes of HCV follow a preferential distribution, since type 1b is most prevalent in Central Asia and Asia Pacific, with Japan and Mongolia being the countries with the highest prevalence. In turn, genotype 3a has been mostly detected in India, Pakistan, Malaysia, Thailand and some regions of Western Europe (109). Genotype HCV-4 is common in the Middle East and Africa, where it is associated with over 80% of infections, but has also spread to Europe. It is also a major cause of chronic hepatitis, liver fibrogenesis and HCC (127). Genotype HCV-6 also increases the risk of HCC, particularly in Asian patients (128).

In patients with post-sustained virologic response (SVR) HCC, regional differences were observed in clinical presentation and prognosis (129). Among 8796 patients with advanced fibrosis (F3/F4) who achieved SVR from 30 sites in Europe, North America, South America, the Middle East, South Asia, East Asia and Southeast Asia, 583 (6.6%) patients developed HCC between 2015 and 2021. Patient outcome varied by region, with a hazard ratio range from 1.82 to 9.92. The best outcomes were in East Asia, North America and South America. The worst outcomes were in the Middle East and South Asia. HCC surveillance was associated with early-stage detection and lower mortality rates. The findings agree with the concept of the “field effect”, whereby despite the eradication of the HCV in the liver and despite a significant decrease in HCC incidence is SVR patients; pre-neoplastic changes persist in some patients, leading to HCC (19). Thus, the persistence of pre-neoplastic changes justifies surveillance in patients having obtained sustained viral response and in particular in those who combine multiple risk factors, some of which may remain after viral clearance, *e.g.*, alcohol misuse, overweight and obesity.

Indeed, the importance of the interaction of multiple risk factors on the emergence of liver cancer requires careful attention. It is highlighted by the evidence that while heavy alcohol misuse alone was associated with an odds ratio of HCC of 4.96; combined exposure to alcohol plus HBV and HCV resulted in an odds ratio of 74.63 (130), this implies that after sustained viral response, the combined effect of alcohol and viral infections may have already generated pre-neoplastic lesions that require surveillance.

### Metabolic disfunction-associated steatotic liver disease

Steatosis is a pathological sign that characterizes a wide spectrum of metabolic liver diseases, frequently associated with obesity or overweight, sedentary life style, alcohol misuse, nutritional imbalance and type II diabetes (131). Steatosis encompasses a spectrum of liver diseases, where more than 5% of hepatocytes show macro-vesicular steatosis, *i.e.*, large fat droplets filling the hepatocyte cytoplasm and displacing the nucleus to the periphery. Depending on how the predisposing risk factors have been grouped to form a syndrome, the nomenclature has evolved in recent years (132).The diagnoses of Nonalcoholic Fatty Liver Disease (NAFLD) and Non-Alcoholic Steatohepatitis (NASH) required exclusion of alcohol misuse. However, the frequent coexistence of steatosis with viral hepatitis, autoimmune diseases, and alcohol misuse led to the proposal of the term metabolic-associated fatty liver disease (MAFLD) in 2020 (133, 134). Three years later, a consensus among experts from 56 countries agreed on the terms Metabolic Dysfunction Associated Steatotic Liver Disease (MASLD) and Metabolic Dysfunction-Associated Steatohepatitis (MASH). These terms were considered less stigmatizing and will hopefully improve the identification of patients with steatosis. The diagnosis of MASLD is based on steatosis (≥ 5% of hepatocytes affected) plus at least one of five cardiometabolic criteria, including body mass index (BMI), insulin resistance, blood pressure, plasma triglycerides, and HDL cholesterol levels, allowing for moderate alcohol consumption (20–50 g/day for women; 30–60 g/day for men) (3, 135). The nomenclature was further redefined in 2024: in case of moderate alcohol intake, the recommended term is MetALD, while ALD is used for cases consuming > 50 g/day (women) or > 60 g/day (men) (136). An important point is, however, that the diagnosis of ALD is defined by a pattern of liver injury occurring in the setting of significant and substantial alcohol consumption, drinking history being a major discriminant factor. Other important diagnostic elements are the presence of alcoholic hepatitis and alcohol-specific markers, such as ethyl glucuronide, phosphatidyl ethanol, and carbohydrate-deficient transferrin (136).

In this review, when referring to studies applying NAFLD and/or NASH diagnostic criteria, we will keep the original terms. However, when referring generically to steatosis plus the criteria mentioned above, we will use the new MASLD/MASH terminology.

Whatever the nomenclature, a continuum of inflammatory activity and variable fibrosis may lead to HCC (137). In this regard, we recently assembled and analyzed a human liver MASLD meta-dataset (n=243) and characterized immune cell infiltrates by deconvolution of transcriptomic data. MASLD and MASH showed enhanced positive immune checkpoint levels, innate immune reactivity and extracellular matrix remodeling. In this series, the expression of fibrogenic markers was correlated with total liver fat area and the inflammation score (138).

In the recent years, MASLD has become a major risk factor, which is frequently associated with varying degrees of chronic inflammation, but not necessarily major changes in liver tissue architecture (139–141). In particular, as described above, MASH creates a chronic inflammatory microenvironment enriched in cytokines, chemokines and growth factors (142) promoting hepatocyte proliferation and retro-differentiation (143), which favors genomic instability, the emergence of preneoplastic lesions and, ultimately, HCCs.

The analysis of the annual percent changes in global burden of liver cancer by etiology from 2010 to 2019 (**Figure 6**) revealed a major impact of MASLD on age-standardized incidence, death and patient disability rates; whereas the impact of alcohol remained stable and that of HCV infection considerably decreased (111). These findings agree with a previous independent study on 532,000 patients showing similar trends (144).

Currently, global prevalence of MASLD is approximately 25% with wide variations around the World (145). For example, in South America and in the Middle East, the prevalence fluctuates around 30%; in contrast to 13% in Africa (57, 146), while in North America, the prevalence lies between 21-24.7% (147). MASLD and MASH are major public health problems in the USA and Western Europe, affecting 80 to 85 million people (148, 149). However, the Asian landscape is more heterogeneous. Population expansion and rural exodus in recent years have led to rates of MASLD ranging from 12.5 to 38% in China (150), 23 to 26% in Japan (151), 27% in Korea (152), 12-51% in Taiwan and 9 to 32% in India (153).

MASLD frequently emerges in the context of sedentary lifestyle, obesity and metabolic syndrome (145, 154). In turn, metabolic syndrome with associated obesity, insulin resistance and type II diabetes, is associated with the emergence of HCC (155). In this context, excessive hepatocyte uptake of triglycerides, decreased β-oxidation of fatty acids and lipid peroxidation lead to production of reactive oxygen species, which trigger hepatic inflammation, fibrogenesis and hepatocarcinogenesis (156, 157).

A subgroup of MASLD patients progress to MASH, characterized by hepatocyte ballooning, inflammation and variable degrees of fibrogenesis (158, 159). Over the recent years, compelling evidence has accumulated to unequivocally demonstrate that MASLD is an increasingly important cause of HCC (160–162). Notably, a substantial proportion of patients with MASLD progress to HCC in the absence of cirrhosis (163).

The impact of MASLD on HCC emergence can be viewed as a dynamic balance with other risk factors, particularly viral infections, throughout the 1980–2024 timeline. Countries with low rates of HBV or HCV infections show a strong association of HCC incidence with MASLD (58, 164, 165). In the United States and in Western Europe, even though HCV is still an important risk factor (166), the incidence of MASLD-related HCC is rapidly and steadily increasing and alcohol misuse remains a major cause (167).

Surprisingly, the Burden of Disease study (1990 to 2019) (137) revealed that MASLD-related liver cancer was not associated with the sociodemographic index. This evidence needs to be interpreted cautiously. It probably results from the multifactorial risk factors leading to MASLD, as suggested by its worldwide increase in incidence. We hypothesize that data acquisition may be affected by suboptimal patients’ self-awareness of at-risk nutritional and physical activity behaviors.

Another variable influencing the impact of MASLD as a risk factor for liver cancer and, in particular HCC, is ancestry. Data from six countries in the ESCALON European-Latin American network prospectively following 429 HCC patients (26) showed that in self-reported non-European patients, MASLD was the most common etiology of HCC (52%); while in self-reported European patients, MASLD accounted only for 15% of HCCs. By contrast, in patients of self-reported European ancestry, the most common etiology of HCC was HCV (38%) (27).

Taken together, the present trend toward an increase in liver cancer, and in particular, HCC incidence in high-income countries seems to result from a combination of Western lifestyle and population aging. The impact of MASLD is predicted to be amplified in the wake of the expected decrease in chronic viral hepatitis, resulting from direct antiviral therapy for HCV and progress in HBV vaccination.

### Age-related risk of HCC

The age spectrum of HCC patients extends beyond that commonly described as an old adult with a longstanding history of pre-existing liver disease. The age of HCC onset is also subject to geographic variations. For example, the BRIDGE study analyzed a total of 18,031 patients with HCC from 14 different countries highlighting differences in the age of HCC onset, being above 60 years in Japan, USA and in Europe, but between 52 and 57 years in China and South Korea (168). Age at diagnosis of HCC is in turn related to the geographic distribution of risk factors (81, 103). In Sub-Saharan Africa, a multicenter study including patients from 14 centers in 7 countries, showed that the mean age at diagnosis was 45 years. Notably, a large number of these patients (49%) were infected with HBV (169). A similar picture was found in 1,541 Peruvian patients, where age at diagnosis was 44 in 50% of patients, with a 71% rate of HBV infection (170). A study of 59,907 patients showed that the country of birth was independently associated with age at the time of HCC diagnosis in the United States, with birth in Sub Saharan Africa and Oceania being strongly associated with early-onset HCC (171).

HCV-related HCC has had a major impact on the “baby boomer” generation (individuals born between 1945 and 1965), that exhibit a notably higher prevalence of HCV infection. This birth cohort effect has led to higher HCC incidence in individuals aged 50–69 years, which led to recommendations on HCV screening in the USA (172).

Increasing age is related to increasing HCC risk of cryptogenic HCCs over HBV-related HCCs. Comparing the 1980–2005 with 2006–2017 periods, the ratio of cryptogenic/HBV HCCs increased from 1:7 to 1:4. Cryptogenic HCCs were detected in older patients, with a lower proportion of male subjects and a higher incidence of smoking and unifocal HCC (173).

An association study between aging-related genes and HCC prognosis revealed a 7-gene score predicting patient outcome. Patients with high scores had HCCs with lower tumor differentiation, higher stage and worse prognosis in both the TCGA and ICGC datasets. The high-risk score was related to metabolism and tumor immunity (174).

One of the age-related molecular pathways may be telomere maintenance. Telomeres are repeated DNA sequences important for chromosomal integrity that shorten during aging as a result of the inactivation of telomerase *(TERT).* The activation of TERT is one of the earliest events in HCC emergence. Aging, liver fibrosis, male sex and excessive alcohol consumption are related to liver telomere shortening (175).

The worldwide burden of cancer is increasing in younger populations. A recent analysis of the Global Burden of Disease (GBD) study between 2010 and 2019 in young adults (15–49 years old) found a global estimate of 78,299 primary liver cancer cases and 60,602 deaths resulting in 2.90 million disability-adjusted years. More than half of the countries worldwide have undergone an increase in incidence rates in young adults with around 12% of deaths due to primary liver cancer occurring in this population. Despite a decline in liver cancer mortality due to most etiologies, MASLD- and alcohol-attributable liver cancer increased by 0.87% and 0.21%, respectively. The highest frequencies of liver cancer incidence, deaths and induced disability were observed in middle SDI countries, but age-standardized death rates attributable to primary liver cancer decreased in high SDI, with an annual percent change of -1.65%. Notably, the highest age-standardized incidence, mortality and disability rates attributable to primary liver cancer were attributable to alcohol misuse. According to this GBD study, alcohol-related liver cancer incidence, mortality and disability were higher by about 6.5-; 6- and 7-fold, respectively, than HBV-, HCV- and MASLD-related liver cancer (176).

As pointed out above in the *Data sources and Methods* section, the validity of the data from the Global Burden of Disease (GBD) study has been questioned because the distinction between primary and metastatic liver cancer is often based on verbal autopsy reports (33). Also, the estimates of incidence, mortality and disability in low SDI areas are based on limited evidence of cause-of-death certification. By contrast, in high SDI areas, a large number of countries provide valid data for primary liver cancer certification. In this context, 2019 data from the WHO mortality database showed age-standardized death rates estimates for Europe and the Americas 30% and 40% lower than those from the GBD study, highlighting the importance of external validation using the IARC cancer registry network and the GLOBOCAN or WHO databases (33).

### Gender-related risk of liver cancer

Gender-related risk of liver cancer is also subject to geographic variations. Globally, liver cancer incidence is 2 to 7 times higher in men than in women (56). However, in Zimbabwe, the male/female ratios are 1.2/1, compared to France with a 5/1 ratio (177). The globally higher incidence of liver cancer in men hypothetically results from their greater risk of exposure to carcinogenic agents (alcohol, tobacco and HBV or HCV infections); whereas in women, estrogens could suppress inflammation (178). In turn, androgens could promote HCC by inducing DNA damage and oxidative stress (179). In line with these findings, recent data indicate that despite being diagnosed at an older age, women with HCC show better survival (180).

### Genetic predispositions

Over most other biological markers, genetic markers provide risk information before clinically detectable disease, remain stable and may not be influenced by the course of liver or intercurrent extrahepatic disease. They also provide information on pathogenic mechanisms. A number of recent studies associate genetic variants with the risk of HCC occurrence in different etiological backgrounds. A retrospective case-control study compared genotype frequencies between HCC cases and HCC-free controls matched for age, sex, HBV and HCV infections. The authors identified polymorphisms predicting individual HCC susceptibility in high-risk HBV and HCV patients, such as *ERCC1, GSTP1, CYP17A1, XRCC3* and *ABCB1.* These findings could contribute to HCC surveillance and early detection (181). Germline HNF1A or G6PC mutations predispose to liver adenomas with potential HCC emergence in a non-fibrotic background. On the other hand, other germline mutations predispose to chronic liver disease, leading to fibrogenesis, cirrhosis and eventually to HCC, for example, hemochromatosis due to HFE mutations, Wilson disease due to ATP7B mutations, alpha-1 antitrypsin deficiency due to SERPINA1 mutations and tyrosinemia due to FAH mutations (182).

The picture is more complex in MASLD, which arises in a backdrop of multiorgan damages linked to obesity and metabolic syndrome. Given the high prevalence of MASLD, it is important to search for biological markers sorting out patients at higher risk of developing chronic liver disease and HCC. Although the bulk of the HCC risk in fatty liver disease relies on chronic inflammation and fibrogenesis, partially determined by genetic variants, about 20% of HCCs arise in non-fibrotic or mildly fibrotic livers in patients with fatty liver disease. In the latter population, genetic polymorphisms have pleiotropic effects on obesity, metabolic syndrome and diabetes, which confound the eventual association between HCC and fatty liver disease. This reason probably explains why there is at present no evidence for genetic variants with major effect size. These findings highlight the relevance of polygenic scores to predict MASLD-related disorders. Therefore, a common strategy is to carry out Genome Wide Association Studies (GWAS) to analyze the association of millions of polymorphisms with disease phenotypes. These gene variants can group into tightly correlated linkage groups. Thus, polygenic models can be used to calculate risk scores using regression models or other refined statistical procedures that associate genetic and non-genetic covariates (183). Notably, polygenic risk scores need to be re-evaluated and, eventually, validated in different geographic settings and populations, for which allele penetrance, xenobiotic exposure and lifestyle could differ, leading to different effect size of the models. Also, given the effect size of polygenic scores and the major effect size of some clinical covariates such as age, sex, and presence of cirrhosis, robust prediction models will require very large discovery and validation cohorts, followed by external validations worldwide (184). With these caveats in mind, robust polygenic scores have been developed to gain insight into the emergence of HCC in a MASLD context and to improve HCC risk stratification. Analysis of European at-risk *versus* general population individuals combined *PNPLA3-TM6SF2-GCKR-MBOAT7* variants in a hepatic fat polygenic risk score adjusted for *HSD17B13* to predict HCC even in the absence of severe fibrosis (185). With a similar approach, a GWAS identified common variants for alcohol-related HCC. A two-stage case-control study was conducted in a cohort of 2107 European patients with alcohol-related liver disease with and without HCC. Data were adjusted for age, sex and liver fibrosis. The authors identified susceptibility alleles in *WNT3A-WNT9A*, which were associated with HCC regardless of liver fibrosis and confirmed previously reported genes associated with alcohol-related HCC risk, namely *TM6SF2, PNPLA3* and *HSK17B13* (186).

## Clinical and epidemiological landscape of liver cancer in different world regions

Global liver cancer incidence and mortality have substantially declined between 2000 and 2020, which is mainly explained by a decrease in incidence and mortality in Southeast Asia, East Asia and Oceania. This steep decline is offset by an increase in Central, Eastern Europe, Central Asia, Latin America and Caribbean and a stabilized plateau in South Asia, North Africa and Middle East. In turn, Sub-Saharan Africa showed a mild decline between 2000 and 2020 (8). These data confirm previous predictions whereby an anticipated decrease driven by the control of HBV and HCV infections was expected to be offset by higher rates of metabolic syndrome (187). However, regional variations in the epidemiological dynamics warrant a closer look at the timeline of liver cancer incidence and mortality in recent years.

### Asia

Taking into account the data from the national cancer sample survey during the years 1998-2007, the incidence of liver cancer was 25.84 per 100,000 individuals, with an age-standardized death rate of 18.82 per 100,000 individuals, given that 80% of the patients have progressed to advanced stages of the disease at the time of diagnosis (188).

Chinese epidemiologists cited viral infection, aflatoxin exposure, water pollution (blue-green algae toxins), excessive alcohol consumption and NAFLD as the most common possible causes (189). However, HBV infection was the most frequent cause with 10% of the general population infected (190), which contributed to the early onset of the disease, since the mean age of diagnosis was about 53 years (191).

In Hong-Kong, the median age was of 68 years for both sexes, with a male: female ratio of 2.7:1. Standardized mortality rates were 13 and 3 per 100,000, for men and women respectively, which was considerably lower than in other parts of the World (192), and the median survival in advanced cases was 11 months (193).

In Japan, 94% of all primary liver cancers were HCCs. Japan showed the highest rates of chronic HBV and HCV infections related to HCC, with most of HCC patients showing positive serology for HCV (about 67.7%), according to the Japan Liver Cancer Study Group (194). However, HCC cases in Japan were most frequently observed in older patients; 63% of HCC patients being over 65 years old (195), an age distribution quite similar to that in Hong Kong.

In other parts of the continent and, in particular, in five Indian cities (Mumbai, Bangalore, Chennai, Delhi, and Bhopal), liver cancer was the fifth most frequent cancer for both genders (196). Age-standardized incidence rates of HCC in India were 0.9–3.4 per 100,000 individuals for men and 0.2–1.8 per 100,000 individuals for women (195), with similar rates in 2022 (197). Among patients with background cirrhosis, the incidence rate increased by 1.6 per 100 person-year and chronic HBV and HCV infections were present in 71% and 16% of patients; respectively, but these data varied from rural to urban areas (198).

In a retrospective analysis of 191 HCC cases (199), the mean age at diagnosis in India was 52 years and the spectrum of clinical presentation overlapped with signs of decompensation of cirrhosis, such as ascites (57%) and gastrointestinal bleeding (22%). In addition, massive hepatomegaly was seen in more than half of the patients (56%), with a mean AFP value of 320 ng/ml. The mean ± SD size of the tumors was 6.8 ± 3.4 cm, with tumors larger than 5 cm in three-fourths of the cases.

More recent reports from India showed high intratumor heterogeneity, with coexistence of different histo-morphological patterns (200). The death rates attributable to liver cancer in deaths per million people have increased from 15.5 in the year 2000 to 23.6 in 2016, with about 1.5-fold increases in HBV- and HCV-related liver cancers and 1.7-fold increase in alcohol-related liver cancer. Notably, the death rates from cirrhosis rose by 1.08-fold within the same time lapse, with a 1.2-fold increase in HBV- and HCV-related cirrhosis and 1.05-fold in alcohol-related cirrhosis. Thus, viral hepatitis remains a major cause of cirrhosis and liver cancer in India (201).

These data imply that liver cancers and, most frequently HCCs, are detected at advanced tumor stages with clinical manifestations and indicate that there is room for implementing surveillance policies for detection and follow-up of patients at risk. In others parts of Asia, such as Philippines (202, 203) and Taiwan (1), HCC is considered among the most lethal oncological diseases and risk factors are the same as those described for the rest of Asia (203–206), except that, in Philippines, patients with cryptogenic causes represent 24.9% of all HCC cases (195).

### Africa

Primary liver cancer in this continent is considered the fourth most frequent neoplasm; however, its prevalence and etiologies present great differences between Northern and Sub-Saharan Africa (SSA) (207). In Northern Africa, the incidence of liver cancer is high, due to the high prevalence of HCV infection (208), with affects over 10% of the general population in Egypt (209, 210), where the median HCC age incidence is 58 years, with a 84% HCV positivity rate. In SSA, where HBV infection remains the major risk factor, 95% of the patients present with advanced or terminal disease (211). The high prevalence of HBV infection leads to a high HCC incidence in patients under 45 years old.

Another very important cause in Africa is exposure to aflatoxin, the most important being B1, derived from *Aspergillus flavus* and *Aspergillus parasiticus* (66). Several studies have shown a potential synergistic effect between aflatoxin B1 and HBV infection (212–214), because the tropical climate favors the proliferation of *Aspergillus* in stored peanuts, corn and other grains for human and animal consumption (215–218).

Notably, iron overload is a risk factor very important in SSA, which is independent from the underlying liver disease. The release of the metal, after the supersaturation of its regulation and storage mechanisms, generates hepatocyte damage due to an increase in free radicals (219). Iron overload in the African diet has been previously described and called Bantu siderosis (220–222). This disease was initially identified in the Central and Southern regions of SSA Africa, mainly in rural areas, where home-made liquor is consumed from sorghum, corn among others; fermented in recycled iron barrels, which had been used mainly for the storage of chemicals. Thus, these liquors contain iron concentrations between 46–82 mg/ml (223, 224).

Lifestyle factors also affect the African population, although obesity and type 2 diabetes occur almost exclusively in urban areas (209). However, in 2019, the prevalence of metabolic syndrome and obesity were, respectively, of 78% and 27% in various SSA countries, associated also with an increase in type 2 diabetes rates (225). SSA is considered the global epicenter of HIV, with HIV and HBV coinfection being common, which leads to the rapid development of the disease and increased lethality (226).

It is important to mention that Uganda has the lowest average age at HCC diagnosis, being 32 years. In addition, 2% of the population affected by HBV-related HCC develops the disease before the age of 20 (227). The usual age of presentation is between 5 and 15 years, with a male-female ratio of 2-3: 1. The clinical manifestations do not differ significantly from those in adults (228). Surprisingly, the fibrolamellar variant, which is typical of young populations, has only been reported in 9% of all cases of HCC in children in SSA (229).

### Central and South America

Approximately 4.8% of liver cancer cases worldwide occur in Latin America. The mean ASIR was 4.8 cases per 100,000 according to GLOBOCAN 2022, which is subject to regional variations (15). In Central America, Guatemala has the highest ASIRs/ASMRs per 100,000 individuals (15.5/14.9), followed by Nicaragua (9.8/9.4), Haiti (9.3/7.8) and Belize (7.0/6.7), with high mortality-to-incidence ratios. This is probably related to nutritional aflatoxin contamination in Central America (230, 231). By contrast, ASIRs/ASMRs are lower in most of South American countries, with Brazil (4.5/4.3), Argentina (3.7/3.1), Perú (4.9/4.2), Colombia (3.6/3.3), Bolivia (6.7/6.0), Venezuela (4.0/3.6) and Chile (4.7/4.5) covering 92% of the surface and 93% of the population of the South American continent. Notably, between the 2010–2019 period, analysis of the WHO database revealed alarming increases in primary liver cancer ASMR in young adults at age 15–49 in some Latin American countries: Mexico (11.5%); Colombia (16.7%); Chile (23%); Uruguay (26.9%) and Argentina (60%) (33).

In relation to risk factors, a first multicentric study, developed in 6 different countries (Brazil, Argentina, Colombia, Peru, Ecuador and Uruguay), pooling information from 14 hospitals and from a total of 1,336 patients, revealed that the main risk factor for the development of HCC was chronic infection by HCV (48%), followed by chronic alcohol misuse (22%), HBV (14%) and NAFLD (9%) (232). More recently, the South American Liver Research Network recently provided a clinical overview of HCC in South America. Although the evaluated cohort is limited to 339 HCC cases diagnosed between 2019–2021 in six countries in South America (Argentina, Perú, Ecuador, Chile, Brazil and Colombia), it is representative of major referral centers. The median patient age was 67 years and 61% were male. The most common risk factors were MASLD (37%), HCV (21%), ALD (17%), HBV (12%), and 13% of the cases were related to unidentified etiologies; 80% of HCC arose in a background of cirrhosis. Notably, HBV-related HCCs occurred in younger patients, with a median age of 46 years. About 27% of the patients received trans arterial chemoembolization or radio-embolization. Only 13% of patients benefited from resection and 6% from liver transplantation, with 9% receiving local HCC ablation, 12% systemic treatment and 17% palliative treatment (233).

A key limitation is that these data are subject to regional variations, as higher rates of HBV-related HCCs have been reported in Perú (34%) and in Brazil (38%) (232). Both countries have the greatest extension in the Amazon basin, known for being an endemic area for HBV infection. Both countries have extensive indigenous communities living in this geographic area.

### The particular case of the Peruvian population

Several studies show that the first cause of HCC in Perú is chronic HBV infection, with a positivity of about 50% (170, 234, 235). HCC patients show a bimodal age distribution with respect to a 44-year-old cut-off (170). A bimodal distribution has also been described in patients from Sub-Saharan Africa (SSA), where younger patients show high prevalence of HBV positivity. However, in SSA, older patients, who represent 84% of all HCCs are predominantly HCV positive (169, 209). In Perú, patients with a negative HBV serology present positive molecular tests for HBV DNA (234). This pattern of occult infection is mainly seen in older patients compared to younger ones. In contrast, the HCV positivity rate in Peruvian patients is between 2 and 4% (170, 234).

An important clinical presentation feature in Perú is tumor size, with a mean diameter of 14 cm and high levels of AFP, with 29,000 ng/ml/tumor-cm in younger and 4,300 ng/ml/tumor-cm in older patients (170, 236). These tumors emerge on a non-cirrhotic liver, which matches the presentation pattern of HBV-related HCC in other regions of the World. An interesting feature in Perú is the presence of lesions in the non-tumor liver of these patients called *“clear cell foci”*, characterized by a clear and abundant Periodic-Acid-Schiff-positive cytoplasm and disruptive architecture with respect to normal hepatic parenchyma, as evidenced by a loss of the reticuline network (235). *Clear cell foci* may probably bear pre-neoplastic potential because they are closely related to the development of HCC in a background of chronic HBV infection.

In a recent study (237), we revealed higher concentrations of toxic metals, such as Cadmium and Arsenic, in HCCs from Peruvian than in those from French patients. Hypothetically, toxic metals could have a pathogenic role in HCC arising in non-cirrhotic livers. Although further studies are required to test this hypothesis, environmental exposure of young Peruvian individuals to toxic metals is unfortunately frequent because of the importance of the mineral industry in Peruvian economy.

### Trends in two high-income countries in Europe and the Americas: the cases of United States of America and France

The USA and France are two high-development-index countries and they both share, according to GLOBOCAN 2022, similarly high age-standardized incidence and mortality rates for liver cancer. Likewise, they both show sub-national geographic and populational heterogeneities in liver cancer presentations and etiologies. In the USA, high population diversity resulting from recent and continued immigration is related to high variations in HCC incidence. In 2001, the HCC incidence in people from Asia and Pacific Islands was the highest (11.3 per 100,000 individuals), but declined by 2.2 points in the following decade. As a result, Alaskan Natives and American Indians had the highest incidence in 2016 (11.4 per 100,000 individuals), while immigrants from Spanish-speaking countries represented 9.8 per 100,100, in contrast with the category described as “non-Hispanic whites” (4.6 per 100,000 individuals) (32). More recently, significant heterogeneities by populational groups were revealed in the incidence of etiology-specific HCCs. Because the USA (in contrast to France) research practice guidelines allow for classification of populational groups by race and ethnicity, ASIRs were used to analyze the intersection between etiology and race-ethnicity in 14,420 clinically documented HCC cases from the Florida cancer registry from 2010 to 2018. Within this period, HCV remained the leading cause of HCC among men, but MASLD became the leading cause of HCC in women since 2017. HCV-related HCCs were high in USA-born minority men (with ASIRs between 7.6 and 10.9 per 100,000 individuals). However, ALD-related HCC remained high among specific “Hispanic” male groups. Since 2015, an increase in the population-based ALD-related HCC rates (+6.0%) and MALSD-related HCC rates (+4.3%) and a rapid decrease of HCV-related HCC rates (-9.6% annually) were observed. Thus, the effect of direct acting anti-HCV treatments on HCV-related ASIRs highlighted the important rise in ALD- and MASLD-related HCCs, particularly in “Hispanic” patients (30). These results were further confirmed by a retrospective cohort analysis of the 31,671 patients diagnosed with HCC in California within the same time frame (2010 to 2018). Patients of Indian, Asian, Pacific Island and Latin American origins were disproportionately affected by HCC. In this cohort, 46% of HCCs were due to HCV, 23% to MASLD, 12% to ALD and 10% to HBV, but by 2017 to 2018, MASLD accounted for 27% of HCCs, confirming the notion that HCV-related HCC decrease is offset by MASLD- and ALD-related progression (31), in line with the data illustrated in **Figure 6** above.

Population-specific inequalities in the USA are also illustrated by a report published in 2021: among 1620 patients, 68% were diagnosed with HCC as outpatients and 32% in emergency departments, the latter presenting more frequently with advanced clinical stages, decompensated disease and aggressive features. In this context, unfavorable predictors applied in the emergency departments at diagnosis were male sex, black skin color, immigrant from Spanish-speaking countries, > 25% below poverty line, uninsured, lack of primary care physician/patient navigator guiding the patient through the healthcare system (238).

Moreover, in a retrospective study of 1117 HCC patients in the USA from 2008 through 2017, 36% had been self-identified as “White”; 34% as “Black” and 30% as “Hispanic”. Patients self-identified as “Hispanic” and “Black” were less likely to be diagnosed with early-stage HCC than those self-identified as “White”. Self-identified “Hispanics” were less likely to benefit from curative treatments than self-identified “Whites”. Moreover, self-identified “Black” and “Hispanic” patients had shorter survival times than self-identified “White” patients (239). Furthermore, a meta-analysis performed in August 2020 from studies on HCC outcomes according to “race and ethnicity” described 563,097 ethnically self-identified patients (53% “White”, 17% “Black”, 18% “Hispanic” and 5% “Asian”). It concluded that “racial and ethnic” disparities had an impact on HCC presentation and prognosis in the US (240).

In France, HCC patients benefiting from early detection and potentially curative treatments show a median survival of 50 months. However, median survival and access to potentially curative treatments in France are subject to regional variations. The French region Brittany has a high HCC incidence, with a median survival of less than 9 months and access to potentially curative treatments in less than 22% of patients (241–243). In a retrospective cohort study of 20,083 incident HCC patients in France between 2015 and 2017, the mean patient age was 69 years and 82% were men. The most frequent etiologies were alcohol-related liver disease in 51%, MASLD/MASH in 44% and viral hepatitis in 20%. Only 33% of patients received curative therapy, with a 1-year survival of 89.5%; 38% of the patients received only best supportive care, with a 1-year survival of 13%. Thus, at least until very recently in France, HCC was still most often diagnosed at an advanced disease stage (244).

## Improving surveillance for at-risk patients

Under sociopolitical contexts that ease the access to the healthcare system, HCC detection is based on screening of patients at risk: chronic hepatitis, MASLD, MASH or other conditions leading to severe liver fibrosis/cirrhosis. In this population, patients with detection of a liver nodule > 1 cm in diameter on abdominal ultrasound imaging and/or serum alpha-fetoprotein > 20 ng/ml are proposed quarterly short-term follow-up. For patients with nodules ≥ 1 cm in diameter, quadruple-phase computed tomography or dynamic contrast enhanced magnetic resonance imaging are proposed. The outcome of a patient with HCC relies on tumor stage (tumor size, number, vascular invasion) and on the underlying liver function. Thus, careful selection of patients for HCC resection with curative intention may reach a 5-year survival ~ 70% for very early- *(i.e.*, BCLC 0) or early- *(i.e.*, BCLC A) stage HCCs, as reported for HCCs ≤ 2 cm (8, 245, 246). This is in contrast with the poor outcome of advanced, symptomatic HCCs, with a median survival about 6 to 18 months. At the individual level, these facts justify follow-up of at-risk patients. Notably, development and implementation of public health screening policies is offset by HCC incidence thresholds in the different categories of at-risk patients to justify cost-effectiveness (5).

As a whole, HCC treatment options are important sources of inequalities because they require infrastructure, logistics, trained personnel and, in view of their cost, the development of performant health insurance policies protecting socially vulnerable populations. For example, in West Africa, curative treatment is impossible, focusing mostly on palliative care. The vast majority of patients diagnosed with HCC enter a medical center in advanced stages of the disease, where focusing on patient comfort is frequently the only option (247). Moreover, there is a clear challenge when implementing palliative strategies; for example, Gambia reports that only 48% of HCC patients receive palliative treatments (248). Improvements in HCC surveillance in the Asia-Pacific region have spurred the adaption of management protocols to the features of the population; in particular, for intermediate stages of the disease, defined as: a single tumor with a maximum size of ≥5 cm or two or three nodules > 3 cm in diameter or > 3 nodules. This strategy prioritizes the preservation of liver function by recommending super-selective conventional TACE with curative intent as first choice of treatment (249).

In South America, despite marked heterogeneity in available resources, health care systems and reimbursement policies, progress has been made in the surveillance of at-risk patients and in the management and treatment of HCC. As described in a multicenter study conducted in 14 hospitals in Argentina (250), treatments applied for advanced HCC were trans-arterial embolization as well as tyrosine kinase inhibitors. In addition, liver transplantation is being routinely and successfully practiced in referral centers applying standardized guidelines (251). Similarly, Brazil has developed its own recommendations for the management of patients with HCC (252) based on Barcelona criteria with some modifications, according to the reality of their country. Although radiofrequency ablation is the treatment of choice for patients with initial stages of the disease and surgery is the treatment of choice for HCC developed on non-cirrhotic liver, liver transplantation procedures have entered routine practice in recent years.

Despite the fact that Argentina has been considered a high HDI country by the IARC (9), a recent study including 301 HCC patients with a mean age of 64, reports that at the time of diagnosis, only 43% of the patients were under HCC surveillance. Among the factors related with the absence (but not the failure of) surveillance on univariate analysis, health care insurance, attention in a private hospital and follow-up by an hepatologist showed significant associations. Among patients with complete surveillance, failure occurred in 25% of the patients; the only variable significantly associated with surveillance dropout was AFP ≥ 20 ng/ml (253). This seminal study suggests that self-awareness of HCC risk factors, if implemented by Public Health policies (media, education), could be determinant and cost-effective in controlling the predicted rise in HCC incidence in South America. Finally, the fact that surveillance of at-risk patients was significantly associated with health care insurance and attention in a private hospital highlights the impact of socioeconomic inequities.

## Conclusion

Primary liver cancers are lethal diseases that have shown an increase in incidence in the recent years. Risk factors as well as patient management strategies vary across the World and are frequently the result of social and economic inequalities. So far, success in patient management relies on screening and early tumor detection in at-risk populations, with curative intent. However, appraisal of tumor heterogeneity and of the mechanisms of cancer progression remain two major challenges to be solved before efficient prediction of liver cancer clinical behavior can routinely be applied across the World. Overcoming these difficulties may help the development of adaptive therapeutic approaches. These challenges could be addressed by the development of cost-effective non-invasive HCC surveillance methods that, in a near future, would allow to appraise tumor heterogeneity and potential for aggressiveness.

The rise in HCC incidence worldwide calls for evolution of HCC risk models to inform surveillance decisions (26, 254). At present, surveillance decisions are currently based on the stage of liver fibrosis and consist on biannual ultrasound plus monitoring serum AFP levels. However, liver fibrosis is not the only determinant of HCC emergence; particularly in the large and ever-growing MASLD population. Thus, alternative HCC screening decisions will need to be based on individual HCC risk, that may be figured out integrating susceptibility factors. The ideal mathematical formula integrating susceptibility factors may include not only etiological, genetic and biological markers assessing liver damage, but also consider environmental and socioeconomic risk factors. The weight of these risk factors drives cancer inequities in Europe (255) and convergent evidence presented here suggests that this is the case worldwide.

## Author disclaimer

The racial and ethnic categories mentioned in this article are cited from external research not conducted in France and are presented here within quotation marks to indicate their origin from the referenced sources. The authors do not endorse the methodology of collecting or processing personal data that reveals the racial or ethnic origin of individuals. This approach aligns with Article 6 of French Law No. 78-17 of January 6, 1978, known as “*Informatique et Libertés*”, which prohibits such practices. Furthermore, we acknowledge the limitations and potential biases inherent in such categorizations and emphasize the importance of interpreting the data with cultural sensitivity and awareness of geopolitical contexts.

## Supplementary material

Supplementary Table 1 Estimated age-standardized incidence, mortality rates and Mortality-to-Incidence ratios (MIRs) of liver and intrahepatic bile duct cancers worldwide per 10^5^ individuals, for both sexes in 2022. Data source: GLOBOCAN 2022, Cancer Today, International Agency for Research on Cancer, World Health Organization (https://gco.iarc.fr/en). Alpha-3 population codes (ISO/UN) and MIR equation are provided.

Supplementary Table 2 Estimated number of deaths and percent increase projections from 2022 to 2050 for liver and intrahepatic bile duct cancer. Estimated timeline of predicted new cases of liver-cancer-related deaths (2025 through 2050) in five continents. Continents, years, predicted numbers, risk change and % are shown. Data sources and processing are described in the legend to **Figure 3**.

Supplementary Table 3 Gross Domestic Product (GDP) *per capita* in current US$ for 181 countries in 2022 and Mortality-to-Incidence ratios (MIRs) of liver and intrahepatic bile duct cancers worldwide, for both sexes in 2022. Data source: GLOBOCAN 2022, Cancer Today, International Agency for Research on Cancer, World Health Organization (https://gco.iarc.fr/en). Alpha-3 codes, population codes (ISO/UN) and MIR equation are provided. For further details on GDP refer to the legend of **Figure 4**.

Supplementary Table 4 Fractions of hepatocellular carcinomas related to different etiologies from 21 World regions (41).
